# Impact of clinical neurophysiological assessment on diagnosis and management of tremor disorders

**DOI:** 10.1016/j.cnp.2025.05.003

**Published:** 2025-06-12

**Authors:** Katherine Longardner, Yasoda Satpathy, Irene Litvan, Dietrich Haubenberger

**Affiliations:** a University of California San Diego, Department of Neurosciences, 9500 Gilman Dr. MC 0886, La Jolla, CA 92093, USA; b University of California San Diego School of Medicine, 9500 Gilman Dr. MC 0886, La Jolla, CA 92093, USA

**Keywords:** Movement disorders, Tremor, Electrophysiology, Neurophysiology, Electromyography, Accelerometry

## Abstract

**Objective:**

To assess the clinical utility of a standardized, non-invasive electrodiagnostic testing protocol in refining the diagnosis and management of patients referred for tremor evaluation.

**Methods:**

In this prospective observational study, patients with tremulous limb movements with indeterminate clinical diagnoses involving tremor as a cardinal symptom were referred by movement disorders neurologists. Participants underwent standardized phenotyping and electrodiagnostic studies for tremor analysis including four-channel surface electromyography polygraphy and two-channel accelerometry.

**Results:**

Clinical and electrophysiological data from 31 consecutive individuals were analyzed. Electrodiagnostic testing refined the differential diagnosis in 25/31 (80.6 %) participants and changed therapy in 14/29 (48.3 %). Changes included adjusting pharmacotherapy (n = 10), undergoing deep brain stimulation surgery (n = 2), or avoiding invasive procedures (n = 2).

**Conclusions:**

We propose that electrodiagnostic testing is a clinically valuable tool that can narrow the differential diagnosis and impact treatment of tremor.

**Significance:**

Clinical evaluation alone may be insensitive in diagnosing the tremor type when findings are subtle or when multiple movement disorders coexist. This may lead to inaccurate diagnosis and management, increasing cost and patient burden, and prolonging or preventing a successful journey towards adequate treatment. Clinical neurophysiology is a useful diagnostic procedure that can detect and quantify movements that may be otherwise indistinguishable by visual observation.

## Highlights

• Non-invasive electrophysiological techniques differentiate hyperkinetic syndromes.
• Electrophysiology can refine the differential diagnosis for indeterminate tremors.
• Neurophysiology can guide treatment decisions for patients with tremor.

## 1 Introduction

Tremors are rhythmic, oscillatory, involuntary movements and are the most common type of movement disorder (Hallett, 2008). The 2018 International Parkinson and Movement Disorder Society consensus criteria for tremor classification categorizes tremor based on clinical features (Axis 1) and etiology (Axis 2) (Bhatia et al., 2018). Clinical features include medical history (e.g., time course, medication exposure), tremor characteristics observed on physical exam (e.g., body part affected, activating conditions), and laboratory tests including electrophysiological diagnostic testing, which is the most accurate way to measure tremor frequency. Many potential underlying etiologies for tremor exist, including medications, metabolic abnormalities, and degenerative diseases. Tremor disorders are typically diagnosed based on visual assessment by a trained specialist. However, the diagnosis may be difficult to determine from physical examination alone when the signs are subtle or when overlapping neurological syndromes are present (Amlang et al., 2020, Jain et al., 2006, Kurtis and Pareés, 2021, Tinazzi et al., 2021). Visual observation has limited ability to discriminate between individual tremor frequencies or change in frequencies within and across tasks. Furthermore, tremors are common, with one population-based study in a large cohort of older adults finding tremor in almost 15 % (Wenning et al., 2005). Tremors may co-exist with other common movement disorders such as Parkinson’s disease and functional neurological symptoms, making it likely that multiple causes of tremor could be present within the same patient. Correct diagnosis of tremor is crucial to avoid unnecessary testing and invasive procedures (Chou et al., 2022) and to provide appropriate treatment, which, depending on the etiology, may include physiotherapy, cognitive behavioral therapy, pharmacotherapy, and procedures such as botulinum toxin injections, deep brain stimulation, or focused ultrasound ablation (Schneider and Deuschl, 2014, Zeuner and Sidiropoulos, 2019).

Electrodiagnostic recordings using non-invasive sensors can measure motion and muscle activity, complementing the physical exam to characterize tremor objectively and quantitatively, with high validity and test–retest reliability (Deuschl et al., 2022, Haubenberger et al., 2016). This procedure can differentiate tremor syndromes or confirm the presence of multiple causes of tremors and can be especially useful in the following scenarios: 1) to diagnose orthostatic tremor (Sander et al., 1998) and to distinguish the following types of hyperkinetic movements: 2) tremor from myoclonus (Everlo et al., 2022, Merchant et al., 2020); 3) enhanced physiological tremor from essential tremor (Vial et al., 2019); and 4) functional tremor from “organic” tremor disorders (Schwingenschuh et al., 2011, Schwingenschuh et al., 2016). While literature exists defining tremor syndromes by electrophysiological features using specific and sensitive “laboratory-based” criteria (Everlo et al., 2022, Gironell et al., 2004, Schwingenschuh et al., 2011, Schwingenschuh et al., 2016), there is limited published evidence about how this technology affects patient outcomes. Here, we describe our center’s experience developing a neurophysiology research protocol for tremor analysis and demonstrate how electrodiagnostic studies can refine the clinical diagnosis and guide therapy for individuals presenting with tremulous movement disorders.

## 2 Methods

### 2.1 Study protocol

The main objective of this prospective observational research protocol was to characterize tremulous movement disorders with indeterminate diagnoses using standardized clinical evaluations combined with electrophysiological techniques. All participants were referred by local movement disorders specialists, including co-author KL. The University of California San Diego Institutional Review Board approved this study (# 191526). Adult participants provided written informed consent. Participants under 18 years of age provided written assent, and written parental permission was also obtained. Included participants were at least two years old and diagnosed with a suspected tremor syndrome (e.g., essential tremor, functional tremor, orthostatic tremor, etc.) by a movement disorders neurologist. Exclusion criteria were any active dermatological condition that would impair attachment of adhesive materials to the skin, such as pemphigus vulgaris or cutaneous infection; any active unstable medical condition, such as unstable angina pectoris, respiratory failure, etc., and inability to provide informed consent. Using the methods described by Vial et al. (2019), we established a standardized protocol for clinical and neurophysiological evaluation of tremor using an existing electromyography (EMG)/nerve conduction study (NCS) machine. Participants provided comprehensive medical history and underwent physical examination, including a detailed movement-disorders focused neurological exam for phenotyping to assess and validate the referring provider’s differential diagnosis. The final diagnosis was determined based on a combination of the clinical exam and electrodiagnostic test findings. Videos of the neurological exam were recorded with written permission. Electrodiagnostic studies using surface EMG and accelerometry were performed as described below, which was typically completed within 40–60 min.

### 2.2 Surface EMG and accelerometry

Participants were seated comfortably upright in a chair. For upper limb recordings, EMG electrodes were placed in a bipolar montage bilaterally over the wrist extensors (extensor digitorum communis) and wrist flexors (flexor carpi ulnaris). An accelerometer was placed on the dorsum of both hands over the third metacarpophalangeal joint. For lower limb recordings (orthostatic syndromes), EMG electrodes were placed in a bipolar montage bilaterally over the vastus lateralis and tibialis anterior muscles, while accelerometers were placed over both patellae. Recording durations were 40 s in each condition, depending on the differential diagnosis of the referring provider, as described in Table 1. Four-channel non-invasive surface EMG polygraphy was used to measure muscle activity at rest and during specific activation tasks, depending on the tremor phenotype. Two triaxial accelerometers (Kistler, sensitivity 20 mV/g) measured the frequency and amplitude of involuntary movements and were recorded in the z-axis. Two amplifiers (Natus Quantum 1280-channel device) were used to record muscle activity. The software was programmed using a standard electrophysiological setup with a screen display sensitivity ranging from 5-20 V/mm, machine gain of 30–50 μV, with a high-pass filter of 10 Hz and low-pass of 200 Hz for EMG channels, and accelerometry using a screen display sensitivity ranging from 5-20 μV, high-pass filter of 0.5 Hz, and a low pass filter of 200 Hz. Standard clinical neurophysiology testing equipment for EMG/NCS (Natus Medical Inc®, Middleton, WI, USA) was used for data acquisition. Tremor was analyzed using an investigational tremor-analysis software package, “CPeak” (Lauk et al., 1999). Parameters obtained from accelerometry included the peak frequency and Half-Width Power, which corresponds to the tremor power under the main spectral frequency peak (Lauk et al., 1999; Raethjen et al., 2004). Quantitative and qualitative analysis of muscle potentials along with spectral and coherence analyses of the EMG signals was performed.

**Table 1:** Recording Conditions According to Differential Diagnosis.

Interpretation of the raw EMG traces and power spectrum analysis data was performed by investigators KL and DH. Tremor syndromes were defined based on the electrodiagnostic test features described in Table 2. A change in diagnosis was defined by an electrodiagnostic test result that either narrowed or expanded the differential diagnosis (or diagnoses) from the referring provider. No treatment was provided during the study; a final report of the electrodiagnostic test results was sent to the referring provider, who discussed the findings with the patient and made clinical decisions regarding therapy. A change in treatment was defined as an initiation or alteration of pharmacotherapy or a decision to either avoid or pursue surgical therapy based on the electrodiagnostic test results. This information was gathered by chart review and correspondence with referring providers.

**Table 2:** Electrophysiological characteristics of hyperkinetic syndromes.

**Essential tremor** (Bhatia et al., 2018, Vial et al., 2019)^a^
• Bilateral upper limb tremor • Frequency 4–12 Hz with a bilateral central component • Frequency reduced by 1 Hz or less during weight-loading • Non-significant coherence between right and left hands
**Enhanced physiological tremor** (Hallett, 2008; Vial et al., 2019)
• Bilateral upper limb tremor with frequency 4 to 12 Hz with an associated EMG spectral peak that is coherent with the mechanical resonant oscillation • Frequency reduces by greater than 1 Hz during weight-loading
**Parkinsonian tremor** (Vial et al., 2019)
• Rest tremor with 4–7 Hz frequency • Re-emergence during posture
**Functional tremor** (Schwingenschuh et al., 2011, Schwingenschuh et al., 2016)
At least three of the following are present: • Tremor paused or decreased in amplitude by half during contralateral ballistic maneuvers • Significant coherence, as defined by two contiguous bins on the coherence plot that rose above the 99 % confidence limit for random coherence at a frequency where there were corresponding peaks in the power spectra • Entrainment or tremor suppression during tapping • Abnormal tapping performance during entrainment
**Dystonic tremor** (Bhatia et al., 2018; Chen and Chen, 2020)
• Tremor syndrome combining tremor and dystonia as the leading neurological signs • Co-contractions of agonist and antagonist muscles
**Task-specific or position-specific tremor**
• Tremor signal quantifiable with a reproducible peak in accelerometry and/or EMG spectra during a specific position or task
**Holmes tremor** (Deuschl et al., 2022; Milanov, 2002, Miwa et al., 1996)
• Frequency 2.5–5 Hz • Large amplitude tremor present at rest, during posture, and kinesis • Proximal and distal rhythmic muscle contractions • EMG burst durations of 150–170 ms with an alternating activation pattern of agonist and antagonist muscles
**Orthostatic tremor (OT)** (Sander et al., 1998, Vial et al., 2019)
**Classical OT** • Bilateral leg tremor when standing with frequency between 13–18 Hz • Significant coherence between right and left legs as defined by two contiguous bins on the coherence plot that rose above the 99 % confidence limit for random coherence at a frequency where there were corresponding peaks in the power spectra **Slow OT** • Bilateral leg tremor when standing with frequency less than 13 Hz • Coherence between legs may or may not be significant
**Myoclonus** (Merchant et al., 2020)
• EMG muscle burst duration ≤ 100 ms
**Orthostatic myoclonus** (Hassan and Van Gerpen, 2016)
• EMG muscle burst duration ≤ 100 ms in the legs when standing

*Note*: This table includes the various hyperkinetic syndromes observed in our cohort.

a Per current understanding, the criteria for essential tremor are also applicable to essential tremor plus.

## 3 Results

**Fig. 1:**
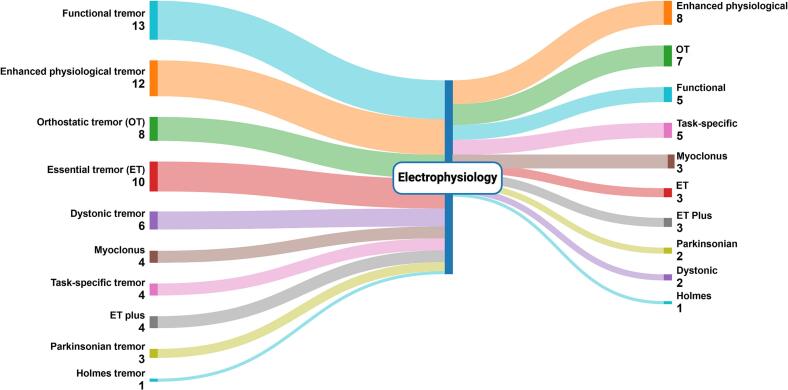
Sankey diagram showing differential diagnosis before electrophysiological tremor analysis and diagnosis after electrophysiological testing in 31 participants referred by movement disorders specialists for evaluation of indeterminate tremor syndrome. Multiple diagnoses may be included for each participant.

We included 31 consecutive participants referred for electrodiagnostic tremor analysis between 2021–2024. Participant demographics and clinical features are shown in Table 3. The median age of participants was 62.0 years (IQR 48.0–75), with 18 (58.1 %) females. The median duration of symptoms was 6.0 years (IQR 3–15). Twenty-one (67.7 %) participants underwent upper limb studies, four (12.9 %) underwent lower limb studies, and six (19.4 %) underwent studies of both upper and lower limbs. Five participants (16.1 %) had clinical features of another movement disorder (e.g., dystonia or parkinsonism). The most common diagnoses on referral (Fig. 1) included functional tremor (n = 13, 41.9 %), enhanced physiological tremor (n = 12, 38.7 %), essential tremor (n = 10, 32.3 %) orthostatic tremor (n = 8, 25.8 %), and dystonic tremor (n = 6, 19.4 %). Less common in the referring providers’ diagnosis were task-specific or position-specific tremor (n = 4, 12.9 %), essential tremor plus (n = 4, 12.9 %) myoclonus (including orthostatic myoclonus, n = 4, 12.9 %), parkinsonian tremor (n = 3, 9.7 %), and Holmes tremor (n = 1, 3.2 %). All but two participants referred had multiple diagnoses included in the differential.

**Table 3:** Demographic and clinical information of participants.

**ID**	**Age (y)**	**Sex**	**Duration of Move-ments (y)**	**Clinical Syndrome**	**DDx for Hyperkinetic Movements**	**Other Movement Disorders**	**Body Parts Studied**	**Electrodiagnostic Tests Diagnosis**	**Electrodiagnostic Results vs. Clinical Diagnosis**	**Change in Diagnosis**	**Change in Treatment**
1	76	F	11	Gait instability and stiffness on standing	Orthostatic tremor	N/a	Lower limbs	Orthostatic tremor	Confirmed	No	Yes; started clonazepam and ultimately underwent bilateral ViM deep brain stimulation surgery
2	48	F	2	Bilateral upper limb tremor with action and at rest and truncal tremor	Enhanced physiological tremor (highest), functional tremor, parkinsonian tremor	N/a	Upper limbs	Parkinsonian tremor	Narrowed	Yes	Yes; started levodopa, which was reported to be beneficial
3	75	F	2	Shaky movements of both hands and left leg with action	Myoclonus, tremor	Atypical parkinsonism	Upper and lower limbs	Orthostatic myoclonus and physiological tremor	Narrowed	Yes	Yes; started clonazepam, which was reported to be beneficial
4	70	F	15	Tremulous movements of legs and imbalance when standing	Orthostatic tremor, confounded by lower back pain and leg paresthesias	N/a	Lower limbs	Orthostatic tremor	Confirmed	No	No; continued clonazepam
5	79	M	21	Bilateral hand action tremor; shakiness/ unsteadiness when standing	Essential tremor plus, enhanced physiological tremor; possible orthostatic tremor but confounded in the setting of multiple comorbidities (peripheral neuropathy, cervical myelopathy, lumbar spinal degenerative disease)	Idiopathic Parkinson's disease (DaTscan positive)	Upper and lower limbs	Orthostatic myoclonus and physiological tremor	Narrowed	Yes	Yes; tried levetiracetam for treatment of movements, but it was not tolerated due to adverse effects
6	56	F	11	Bilateral upper limb action tremor	Enhanced physiological tremor, essential tremor	N/a	Upper limbs	Enhanced physiological tremor with a central component (essential tremor)	Confirmed	No	No; declined pharmacotherapy
7	43	M	10	Unilateral upper limb action tremor	Task-specific tremor, functional tremor	N/a	Upper limbs	Posture-specific tremor	Expanded	Yes	No; declined pharmacotherapy
8*^c^*	44	M	2	Unilateral upper limb tremor at rest and with action	Functional tremor (highest), parkinsonian tremor	Parkinson's disease	Upper limbs	Parkinsonian tremor	Narrowed	Yes	Yes; increased trihexyphenidyl, which was reported to be mildly beneficial, patient is considering focused ultrasound ablation or deep brain stimulation
9	80	F	8	Bilateral upper limb tremor with action; bilateral leg tremor when standing	Orthostatic tremor, orthostatic myoclonus	Dystonic head tremor	Upper and lower limbs	Slow orthostatic tremor	Narrowed	Yes	Yes; increased clonazepam
10	80	M	39	Bilateral leg discomfort when standing and postural instability	Orthostatic tremor, orthostatic myoclonus, possible dystonic posturing of lower limbs	N/a	Lower limbs	Orthostatic tremor	Narrowed	Yes	Yes; continued gabapentin, titrated off propranolol
11	13	M	2	Tremulous movements of both upper limbs with action	Myoclonus, tremor	N/a	Upper and lower limbs	Myoclonus	Narrowed	Yes	No; continued clobezam and levetiracetam for seizures/myoclonus
12	42	F	5	Unilateral upper limb tremor at rest and with action,	Holmes tremor, query dystonic tremor component	N/a	Upper limbs	Holmes tremor	Confirmed	No	No; underwent unilateral Vim and GPi deep brain stimulation surgery (already planned)
13	62	F	6	Bilateral upper limb action tremor	Essential tremor, query component of functional or dystonic tremor given marked asymmetry	N/a	Upper limbs	Dystonic tremor, could not exclude a component of functional tremor	Narrowed	Yes	Yes; avoided deep brain stimulation surgery, referred to occupational therapy, which was reported to be beneficial
14	44	F	4	Bilateral upper limb action tremor; bilateral lower limb tremor	Enhanced phyiological tremor, essential tremor; orthostatic tremor	N/a	Upper and lower limbs	Orthostatic tremor	Narrowed	Yes	No; declined pharmacotherapy
15	36	M	2	Bilateral upper and lower limb tremor at rest and with action	Functional tremor	N/a	Upper limbs	Functional tremor	Confirmed	No	No
16	54	F	13	Unilateral upper limb action tremor	Essential tremor, parkinsonian tremor, dystonic tremor	N/a	Upper limbs	Task-specific tremor	Expanded	Yes	No; declined pharmacotherapy
17*^b^*	71	F	16	Bilateral upper limb action tremor	Enhanced physiological tremor, essential tremor plus	N/a	Upper limbs	Essential tremor plus (questionable dystonic posturing) and enhanced physiological tremor	Confirmed	No	Yes; started primidone, which was reported to be beneficial
18	67	M	3	Bilateral upper limb action tremor	Enhanced physiological tremor, essential tremor	N/a	Upper limbs	Enhanced physiological tremor	Narrowed	Yes	No; declined pharmacotherapy
19	64	F	13	Bilateral upper limb action tremor; bilateral leg tremor when standing	Enhanced physiological tremor, essential tremor; orthostatic tremor	N/a	Upper and lower limbs	Orthostatic tremor and enhanced physiological tremor	Narrowed	Yes	Yes; increased clonazepam
20*^a^*	60	M	2	Unilateral hand action tremor after a stroke	Functional tremor, dystonic tremor	N/a	Upper limbs	Functional tremor	Narrowed	Yes	No
21	82	F	5	Unilateral hand action tremor	Primary writing tremor, dystonic tremor, functional tremor	N/a	Upper limbs	Task-specific tremor	Expanded	Yes	Yes; underwent unilateral ViM deep brain stimulation surgery
22	69	M	3	Asymmetrical bilateral hand action tremor	Enhanced physiological tremor, essential tremor, dystonic tremor	N/a	Upper limbs	Position-specific tremor and enhanced physiological tremor	Narrowed	Yes	No; declined pharmacotherapy
23	59	F	20	Bilateral leg tremor when standing	Orthostatic tremor, functional tremor	N/a	Lower limbs	Slow orthostatic tremor	Narrowed	Yes	No; declined pharmacotherapy
24	59	F	51	Bilateral hand action tremor and larger amplitude unilateral hand tremor much worse when writing	Essential tremor plus, enhanced physiological tremor, primary writing tremor, dystonic tremor	Primary writing dystonia	Upper limbs	Position-specific tremor	Narrowed	Yes	No; declined pharmacotherapy
25	57	M	3	Bilateral upper limb action tremor	Enhanced physiological tremor, essential tremor	N/a	Upper limbs	Essential tremor	Narrowed	Yes	Yes; treated with propranolol
26	20	M	15	Bilateral upper limb tremulous movements with action	Enhanced physiological tremor, essential tremor, functional tremor, myoclonus	N/a	Upper limbs	Functional tremor and enhanced physiological tremor	Narrowed	Yes	Yes; recommended counseling or hypnosis; no additional medications trialed
27	55	F	5	Unilateral hand tremor	Position-specific tremor, functional tremor	N/a	Upper limbs	Functional tremor	Narrowed	Yes	No
28	81	M	9	Bilateral upper limb action tremor	Essential tremor, functional tremor	N/a	Upper limbs	Essential tremor	Narrowed	Yes	No; propranolol continued, offered dose increase but patient declined
29	89	M	6	Right upper limb tremor at rest and with action	Functional tremor, parkinsonian tremor	Idiopathic Parkinson's disease (DaTscan positive)	Upper limbs	Functional tremor	Narrowed	Yes	Yes; avoided focused ultrasound ablation surgery
30	71	F	15	Bilateral upper limb action tremor and unilateral upper limb rest tremor	Essential tremor, functional tremor	N/a	Upper limbs	Essential tremor plus (rest tremor)	Expanded	Yes	N/a
31	74	F	6	Bilateral upper limb action tremor	Dystonic tremor, functional tremor	Parkinsonism (suspect drug-induced) and cervical dystonia	Upper limbs	Essential tremor plus	Expanded	Yes	N/a

**Abbreviations and symbols**.

Dx – diagnosis; DDx – Differential diagnosis; EP – electrophysiological.

*a* – Case 1; *b* – Case 2; *c* – Case 3.

Clinical neurophysiological assessment refined the differential diagnosis in 25/31 cases (80.6 %), narrowing the diagnosis in 20 (64.5 %) participants and expanding the diagnosis in five (16.1 %). In 23 participants (74.2 %) the electrodiagnostic results were consistent with a single diagnosis and in eight participants (25.8 %), electrodiagnostic results were consistent with two distinct diagnoses. Electrodiagnostic tests revealed enhanced physiological tremor in eight (25.8 %) participants, which often co-occurred with other tremor types. The next most common tremor type characterized by electrodiagnostic tests in this cohort was orthostatic tremor (n = 7, 22.5 %), functional tremor (n = 5, 16.1 %), task-specific or position-specific tremor (n = 5, 16.1 %), essential tremor (n = 3, 9.7 %), essential tremor plus (n = 3, 9.7 %), myoclonus (n = 3**,** 9.7 %), parkinsonian tremor (n = 2, 6.5 %), and Holmes tremor and dystonic tremor (n = 1, 3.2 % for both). The electrodiagnostic test results changed therapeutic management in 14/29 (48.3 %) individuals, with follow-up data missing for two participants. Among the 15 cases in which treatment was unchanged, eight participants were offered pharmacotherapy but declined, three continued their existing medications, and three patients with suspected functional tremor had confirmation of the suspected diagnosis and were not prescribed additional pharmacological treatments. One participant was already preparing to undergo deep brain stimulation surgery, and after confirming with electrodiagnostic tests that the dominant involuntary movement was Holmes tremor rather than dystonia, she underwent surgery as planned. Among the cases in which the electrodiagnostic test results altered treatment, verifying the clinical diagnosis often led to escalation of therapy, including increase of medication dose or initiation of a new medication (n = 10), or pursuance of deep brain stimulation surgery (n = 2). The findings from the electrodiagnostic tests discouraged two other patients from undergoing an invasive procedure. The following three case examples illustrate the impact of EMG and accelerometry on diagnosis and management of patients presenting with indeterminate tremor syndromes.

### 3.1 Case 1

A 60-year-old right-handed man presented for evaluation of right-hand tremors that started after a stroke. His medical history was notable for diabetes mellitus, hypertension, hyperlipidemia, and heart failure due to ischemic heart disease, status post pacemaker and automatic implantable cardioverter defibrillator placement. Two years prior, he suffered an ischemic stroke, presenting with lightheadedness followed by acute right hemiparesis. Head CT showed no acute findings, but CTA showed high-grade left ICA stenosis. He underwent emergent left carotid stenting and recovered 90 % within 48 h, returning to baseline except for mild right-hand weakness. Three months later, he developed an intermittent action tremor in his right hand, which progressively worsened and interfered with daily activities. Gabapentin 300 mg BID was ineffective. A brain MRI 18 months post-stroke showed no ischemic sequelae. Aside from the tremor, his exam revealed give-away weakness in his right arm without other focal findings.

**Fig. 2:**
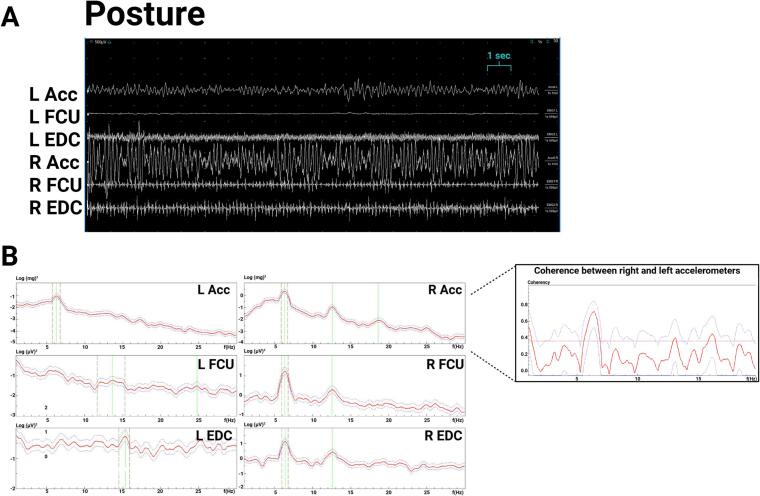
**Case 1 electromyography (EMG) and accelerometry recording from bilateral upper limbs during posture, 2.5-Hz tapping task (entrainment), and contralateral ballistic maneuvers. 0.2A**. EMG recording during posture shows rhythmic movements and alternating muscle bursts in the right hand. **2B.** Spectral analysis shows a 6.3 Hz right hand tremor and a small peak in the left-hand accelerometer that is likely transduction artifact from the large amplitude right hand tremor, since no tremor was observed clinically, and coherence was significant between both right and left accelerometers at ∼ 6.3 Hz but not significant between the right and left EMG.. Abbreviations: EMG: electromyography; L: Left; R: Right; Acc: accelerometer; FCU: flexor carpi ulnaris; EDC: extensor digitorum communis.

**Fig. 3:**
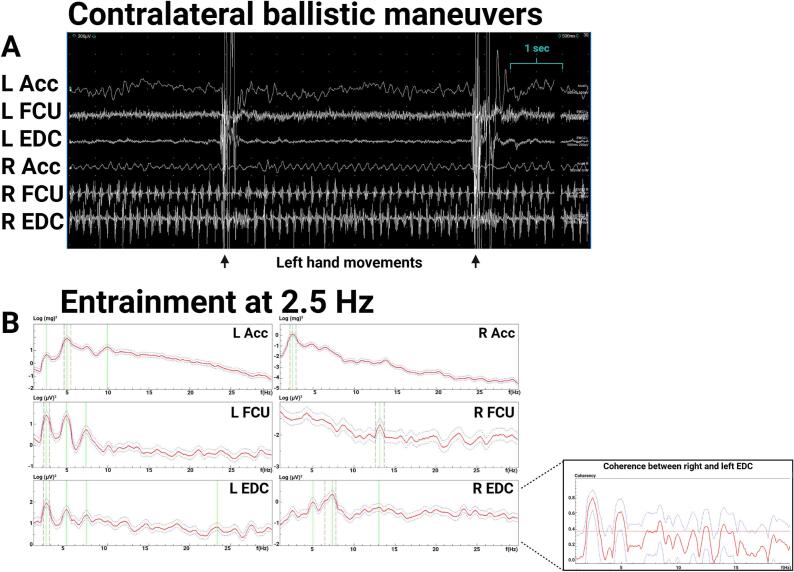
**Case 1 electromyography (EMG) and accelerometry recording from bilateral upper limbs during contralateral ballistic maneuvers and during 2.5-Hz tapping task (entrainment). 3A.** EMG recording shows attenuation of right hand tremor (in posture) with left hand ballistic maneuvers. **3B.**Spectral analysis of recording during entrainment task with right hand in posture and left hand tapping at 2.5 Hz shows a dominant frequency in the left hand at 2.5 Hz and dominant frequency in the right-hand accelerometer with EMG correlate at 2.5 Hz, demonstrating entrainment. The right-hand tremor amplitude is lower, demonstrating distractability. Coherence analysis between right and left EDC shows significant coherence at 2.5 Hz. Abbreviations: EMG: electromyography; L: Left; R: Right; Acc: accelerometer; FCU: flexor carpi ulnaris; EDC: extensor digitorum communis.

Exam (Video 1) showed a 1–3 cm right-hand tremor at rest and during posture and a 1–2 cm kinetic tremor with both hands during finger-to-nose testing. The right-hand postural tremor was distractible, with pause during contralateral hand movements. Electrodiagnostic testing showed a 6.3-Hz peak in the right-hand accelerometer during posture with EMG correlate, and a small peak around 6 Hz on the left-hand accelerometer (Fig. 2A, 2B), which was interpreted as transduction artifact since the bilateral accelerometer, but not bilateral EMG, showed significant coherence at this frequency, and no tremor was observed in the left hand during this recording. During left hand ballistic movements, there was a pause in the right-hand tremor in most of the traces (Fig. 3AC), demonstrating distractibility. During entrainment at 2.5-Hz (left FCU frequency 2.5 Hz) while the right arm is extended in posture, the right accelerometer showed a dominant frequency of 2.5 Hz, with EDC correlate (Fig. 3B), and the tremor amplitude reduced. During entrainment tasks, there was significant coherence between the right and left EDC EMG. The entrainment frequency at 2.5 Hz was chosen since it is sufficiently distinct from the patient’s tremor frequency and the patient’s tremor frequency does not represent a multiple of the tapping frequency. These results were consistent with a functional tremor, and the patient was referred to occupational therapy. While dystonic tremors have been reported with thalamic strokes (Gupta and Pandey, 2018) there was no neuroimaging evidence of a thalamic lesion, and no dystonic posturing was observed.

### 3.2 Case 2

A 71-year-old right-handed woman presented with about 16 years of bilateral action tremor of the hands that started gradually. She first noticed the tremor when using power tools or hammering. The left hand was more affected than the right. Over the years, her hand tremors became more noticeable, impairing her writing and her ability to apply makeup and to eat with cutlery. She had no family history of similar tremor, but two maternal uncles had Parkinson’s disease. Her medical history was notable for depression, which was treated with escitalopram and bupropion. She habitually drank two to three cups of coffee daily and did not consume alcohol. She previously took propranolol 40 mg in the morning and 20 mg at nighttime for paroxysmal atrial tachycardia. She did not try higher doses due to side effects of fatigue. Propranolol initially helped her tremor, but its beneficial effect waned within a year, and it did not help her tachycardia. She switched to atenolol, which also was not helpful for her tremor. Gabapentin 600 mg twice daily had only minor benefit. The differential diagnosis was enhanced physiological tremor versus essential tremor plus. Given her age, her neurologist was hesitant to try other medications such as primidone without being more certain of the tremor type.

**Fig. 4:**
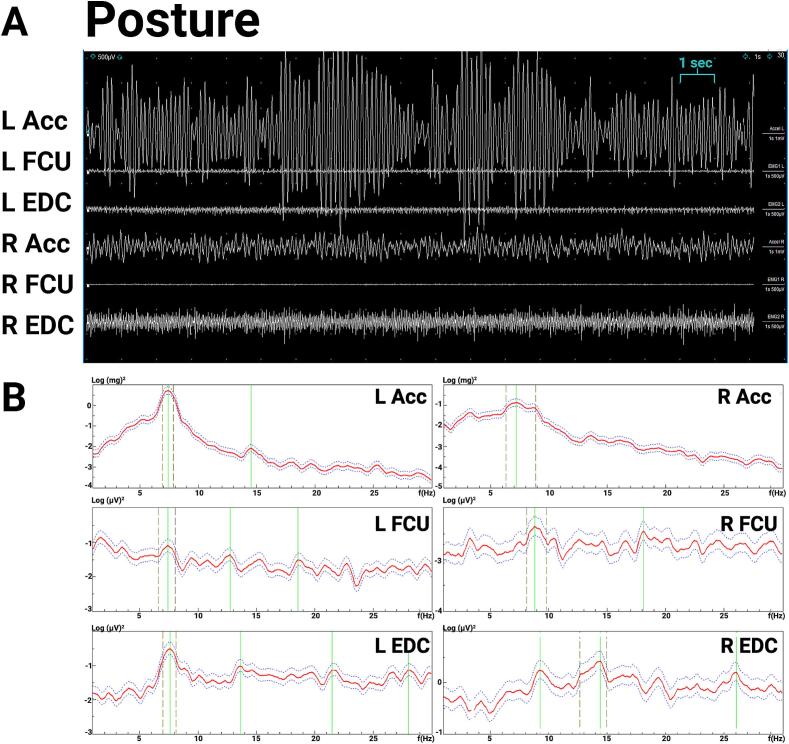
**Case 2 electromyography (EMG) and accelerometry recording from bilateral upper limbs during posture. 4A.** EMG recording. **4B.** Spectral analysis. During posture, both accelerometric frequencies were about 7 Hz, with EMG correlate (left hand about 7 Hz, right hand about 9 Hz). The amplitude of the left-hand tremor was 1–2 times greater than the right. Abbreviations: EMG: electromyography; L: Left; R: Right; Acc: accelerometer; FCU: flexor carpi ulnaris; EDC: extensor digitorum communis.

**Fig. 5:**
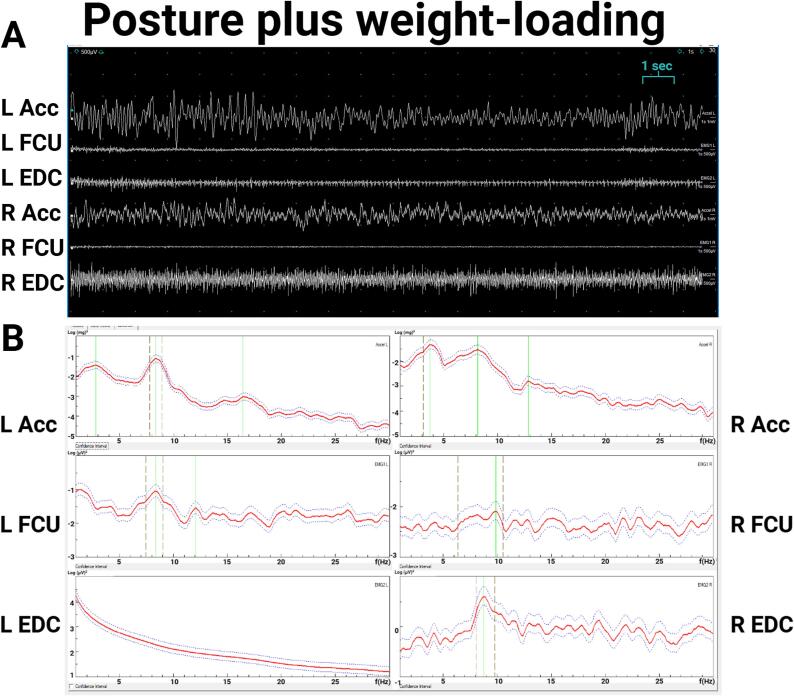
**Case 2 electromyography (EMG) and accelerometry recording from bilateral upper limbs during posture with weight-loading. 5A.** EMG recording. **5B.** Spectral analysis. During weight loading with 1.5 lbs. during posture, the left-hand accelerometer showed a 4.1-Hz mechanical component without EMG correlate and an 8.3 Hz central component with EMG correlate, and the right-hand accelerometer showed a 3.8 Hz mechanical component without EMG correlate and an 8.9 Hz central component with EMG correlate. The left EDC spectrum is artifact. Abbreviations: EMG: electromyography; L: Left; R: Right; Acc: accelerometer; FCU: flexor carpi ulnaris; EDC: extensor digitorum communis.

The exam was notable for a 1–2 cm postural and kinetic tremor in both hands, with a larger amplitude on the left side. There was mild abnormal posturing in the left fingers, which may have been related to a prior hand injury (Video 2). Electrodiagnostic testing (Fig. 4) demonstrated a ∼ 7–8 Hz centrally generated action tremor in both hands, with greater amplitude in the left hand, with an additional peripheral mechanical reflex component (Fig. 5). Given the asymmetry of the tremor and questionable abnormal posturing of left fingers on exam that was possibly related to her history of peripheral injury, this was consistent with “essential tremor plus”. In addition, a peripheral component of a physiological tremor was observed on electrodiagnostic tests, with caffeine and medications such as escitalopram and bupropion likely contributing to the latter. Based on these results, through shared decision making, she and her neurologist decided to start primidone for symptomatic treatment, which was reported to be beneficial at doses of 250 mg daily. The propranolol that she initially tried may have been effective for the peripheral component of her tremor, whereas later, the primidone was more helpful for the central component.

### 3.3 Case 3

A 44-year-old right-handed man presented with 20 months of right-hand tremor. He initially noticed tremor intermittently when using his hand to do activities and it became more obvious whenever he experienced a strong emotion, e.g., anxiety or anger. About five months after onset, the tremor began to occur daily both at rest and with willed action, intensifying during public speaking and periods of strong emotion. The tremor impaired his ability to prepare food, write, shave, brush his teeth, drink from a glass, type, text, and work, which involved using tools to cut wood. One year after tremor onset, he sought neurological evaluation and was diagnosed with Parkinson’s disease. He denied dysphagia, voice changes, other involuntary movements, imbalance, problems with manual dexterity, gait changes, falls, cognitive changes, hallucinations, sleep disturbances, history of dream enactment, orthostatic symptoms, constipation, urinary symptoms, or hyposmia. His medical history was only notable for hypertension, treated with lisinopril/hydrochlorothiazide. He was not taking any other medications or supplements and did not consume alcohol, caffeine, drugs, or tobacco. His father was 66 years old and had a mild hand tremor. There was no other family history of neurological disorders in his two siblings or three children. He tried carbidopa/levodopa 25/100 mg instant release and titrated up to two tablets TID, but this had no perceived benefit on his tremor, and he had side effects of headache and nausea, so it was discontinued. He tried amantadine 100 mg BID, but this had no perceived benefit and caused nausea, so it was also discontinued. Trihexyphenidyl 1 mg BID reduced the tremor amplitude modestly.

Exam showed a constant large amplitude right-hand rest tremor that persisted with posture and kinesis and was not distractible or entrainable. He had slight right-sided parkinsonism, with rigidity in the right upper limb present only with contralateral activation maneuvers, and slight slowing during right hand rapid alternating movements (Video 3). Otherwise, the neurological exam was normal. Brain MRI was normal and DaTscan showed mildly decreased uptake bilaterally, greater on the left than the right. Although the patient’s exam showed minimal parkinsonian signs and DaTscan supported a disorder of dopaminergic deficiency, his lack of response to levodopa, young age, and tremor characteristics, including presence during action and short latency from rest to posture, raised suspicion for a functional tremor or functional overlay.

**Fig. 6:**
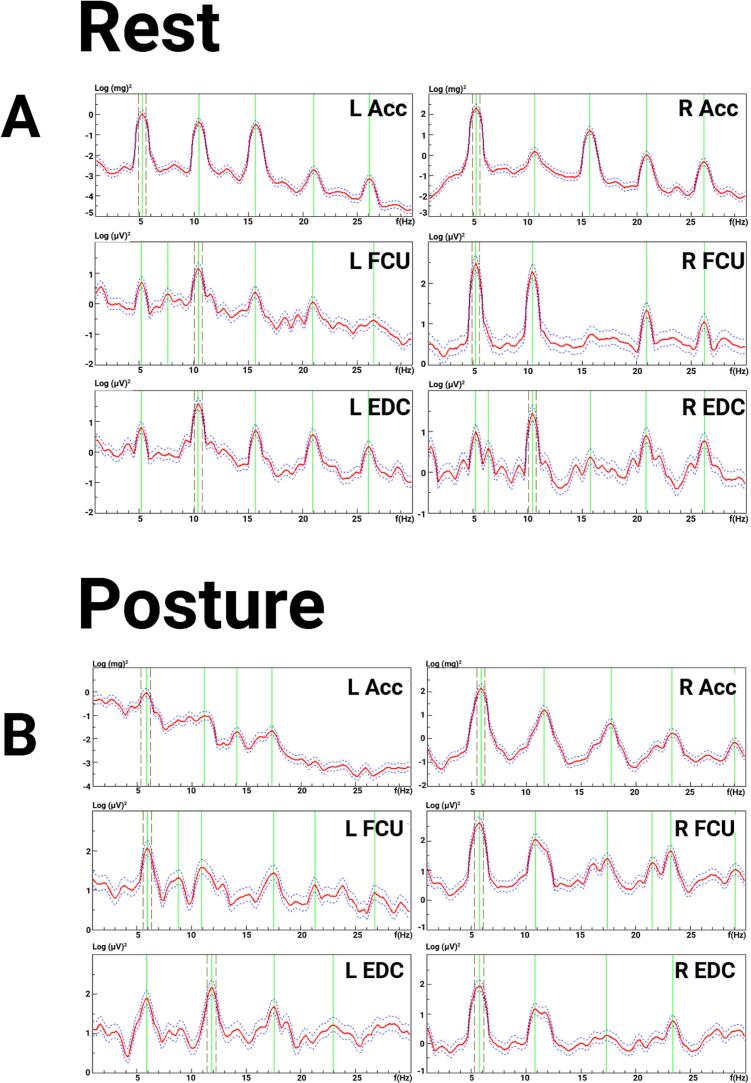
**Case 3 electromyography (EMG) and accelerometry recording from bilateral upper limbs at rest and during posture. 6A**. Spectral analysis showed a 5.2 Hz rest tremor in the right hand. A 5.2 Hz tremor signal was also seen on accelerometry and surface EMG recordings in the left hand, but no tremor was observed visually. This signal detected from the left upper limb was most likely due to motion artifact from the large amplitude right upper limb tremor. **6B.** During posture, there was a right-hand tremor with 5.9 Hz frequency. The left-sided accelerometer and EMG tracings showed a 5.9 Hz signal. Again, no tremor was observed visually in the left upper limb, so this signal from the left upper limb was most likely due to motion artifact. Abbreviations: EMG: electromyography; L: Left; R: Right; Acc: accelerometer; FCU: flexor carpi ulnaris; EDC: extensor digitorum communis.

**Fig. 7:**
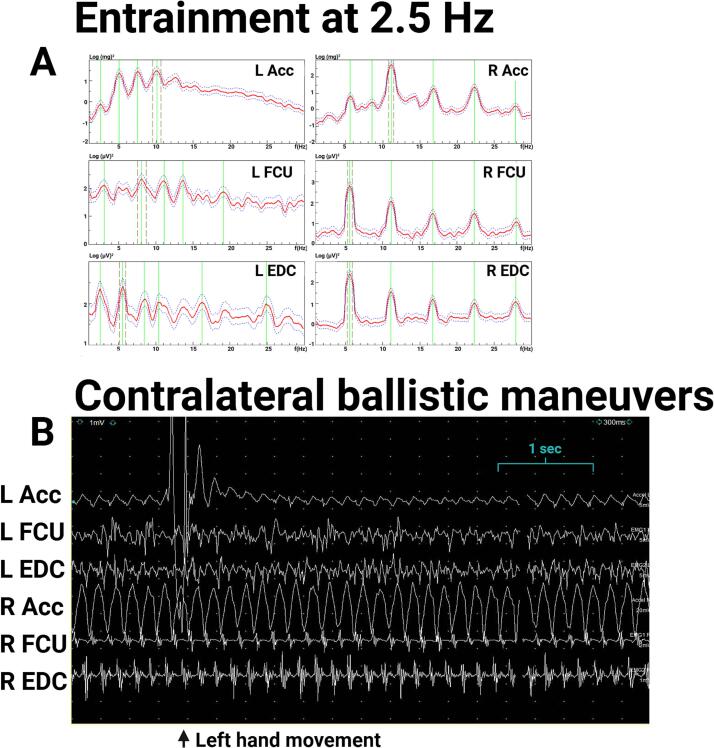
**Case 3 electromyography (EMG) and accelerometry recording from bilateral during 2.5-Hz tapping and contralateral ballistic maneuver. 7A.** During entrainment maneuvers with the left hand tapping at 2.5 Hz, the right-hand tremor frequency remained 5.6 Hz. **7B.** There was no change in the right-hand tremor during contralateral ballistic maneuvers. Abbreviations: EMG: electromyography; L: Left; R: Right; Acc: accelerometer; FCU: flexor carpi ulnaris; EDC: extensor digitorum communis.

Electrodiagnostic testing (Fig. 6) showed a stable right-hand tremor at rest (6A) and with posture (6B), with frequency ranging from 5.2 to 6 Hz. There were no electrophysiological features of a functional tremor (i.e., entrainability (Fig. 7A) or pause with dual task maneuvers (Fig. 7B)). This was consistent with a centrally driven tremor, diagnosed as parkinsonian given the phenotype and neuroimaging findings. After these results, his provider increased trihexyphenidyl from 2 mg TID to 3 mg TID, which provided mild additional benefit, although did not resolve the tremor. He was encouraged to explore advanced treatment options given non-levodopa responsive disabling tremor in his dominant hand and at the time of writing this manuscript is considering focused ultrasound ablation.

## 4 Discussion

Our results from 31 patients referred for electrodiagnostic testing support the impact of this technology in clarifying the diagnosis of clinically indeterminate tremor syndromes and directing therapeutic management. Electrodiagnostic testing refined the diagnosis in 81 % (n = 25) of participants. Given validated “laboratory-based” criteria for diagnosing functional tremor based on neurophysiology (Schwingenschuh et al., 2011, Schwingenschuh et al., 2016), this procedure was especially helpful in distinguishing functional tremor from “organic” tremor syndromes. Fourteen (45.2 %) of the participants were referred with a differential diagnosis that included functional tremor. Among these, only five had electrodiagnostic test results consistent with functional tremor. Electrodiagnostic tremor analysis was also beneficial to narrow the diagnosis among participants who had multiple types of tremor-like movements, which may have been difficult to distinguish by clinical observation alone. In our cohort, seven participants (23.3 %) had electrodiagnostic evidence of physiological tremor co-occurring with another type of tremor (e.g., position-specific tremor, functional tremor, essential tremor). Of note, we separated essential tremor and essential tremor plus into two different diagnostic categories. We acknowledge that this distinction is based on clinical information (Bhatia et al., 2018) rather than neurophysiology, although recent evidence suggests that these tremor syndromes share similar electrophysiological characteristics (Rajan et al., 2024).

The electrodiagnostic tremor analysis changed treatment in nearly half (n = 14) of participants. Among the 15 participants who did not alter therapy based on the clinical neurophysiology findings, many were reassured that their tremor syndrome was not due to a progressive or degenerative disorder (e.g., in the cases of enhanced physiological tremor or task-specific tremor) and declined medication for symptomatic treatment. In some cases of clinically overt functional tremor, electrodiagnostic testing provided objective evidence that helped convey the findings to patients who were initially reluctant to accept this diagnosis. In general, the electrodiagnostic test results provided objective data that allowed for greater diagnostic certainty, which guided providers’ confidence in risk/benefit discussions regarding medication adjustment for symptomatic treatment. In this context, clinical neurophysiology procedures can be especially helpful when an unclear diagnosis causes reluctance to initiate or increase medications that have potential for adverse side effects such as sedation (e.g., clonazepam, primidone). Case 2 illustrates this: electrodiagnostic testing confirmed a centrally driven tremor (essential tremor plus) with a peripheral component. These findings led the patient to trial primidone, which effectively controlled her tremor.

Confirmation of the diagnosis can also allow providers to fully exhaust second and third-line therapeutic options and access experimental treatments, and to feel more comfortable exploring invasive surgical options after confirming an organic movement disorder. For example, regarding a patient who was initially suspected to have functional tremor and had electrodiagnostic test results consistent with essential tremor plus, a referring provider reported, “having the results of this makes me feel more comfortable exploring things like deep brain stimulation”. In our cohort, two participants (one with orthostatic tremor and one with task-specific tremor) underwent deep brain stimulation surgery after confirming their diagnoses with electrodiagnostic testing. Case 3 highlights this point, where electrodiagnostic test results from a participant originally suspected to have functional tremor showed a central tremor without any features suggestive of functional tremor. After receiving these results, trihexyphenidyl was increased, which was mildly helpful. Two years later, at the time of writing this manuscript, his tremor-dominant parkinsonism progressed. He continues to take trihexyphenidyl 3 mg BID and has experienced modest improvement with carbidopa/levodopa extended-release capsules. He is considering undergoing focused ultrasound ablation or deep brain stimulation surgery.

Two participants avoided invasive procedures based on the electrodiagnostic test results. A participant with both functional tremor and Parkinson’s disease decided not to pursue focused ultrasound ablation for his right-hand tremor when the electrophysiology findings suggested that his tremor was functional – he instead decided to try hypnotherapy. Another participant who was diagnosed with essential tremor was preparing to undergo deep brain stimulation surgery, but after electrodiagnostic test results indicated dystonic hand tremor with a possible functional component, she deferred surgery and instead tried occupational therapy, which was beneficial.

Our data supports the diagnostic value of clinical neurophysiology procedures for tremor disorders, similar to findings from previously published retrospective studies in larger cohorts from tertiary referral academic centers, which have demonstrated that neurophysiology can refine the diagnosis of tremulous movement disorders (Everlo et al., 2022, Grippe and Chen, 2023, Jackson et al., 2021). Strengths of our research include prospective data collection using a standardized protocol with well-characterized participants and follow-up data on the clinical outcomes. Our electrodiagnostic test setup containing two accelerometers and four-channel EMG was chosen to support the majority of clinical tremor analyses, allowing also lower limb and bilateral tremors. The advantage of this setup is that it can be deployed with standard clinical diagnostic EMG/NCS machines and does not require the acquisition of specialized equipment. We acknowledge that in the research setting, additional spatial accelerometric and gyroscopic axes may be required to fully describe the motion of a limb in space for assessment of hyperkinetic movements (a triaxial accelerometer and triaxial gyroscope). However, for clinical diagnostic purposes, particularly for tremor and tremor-like movements, a single-axis accelerometer aligned with the primary axis of oscillation is largely deemed sufficient. This is especially true for uni-axial movements, such as tremors that involve flexion/extension patterns. A deliberate strength of our approach is the use of a minimal sensor setup that is simple to implement (and can be completed in under an hour) yet sufficient to capture the dynamics of agonist and antagonist muscles across a single joint axis. Using this setup, a variety of tremor syndromes (e.g., proximal, asymmetric, focal, etc.) can be assessed. However, since our electrophysiological study was biased toward recording tremulous movement disorders and not all types of hyperkinetic movement disorders, this may have led to missing cases of myoclonus, which is a limitation of our study. Thus, differential diagnosis of myoclonus was outside the scope of this analysis, and for comprehensive workup of myoclonus, additional electrodiagnostic testing methods may be necessary (e.g., using somatosensory stimuli, EEG, combined EEG/EMG analyses, etc.). Other limitations of our study include a relatively small sample size and potential for referral bias, as most patients were referred from movement disorders specialists at our institution. Although many tremor analysis systems have demonstrated high validity and test–retest reliability, we acknowledge that there are no standardized techniques for tremor analysis with established software or identical commercial systems. Each unique electrodiagnostic set-up requires calibration and normative data generated with that system for proper comparisons. Consequently, the analysis method may influence data interpretation. For example, the spectral plots that we included represent averages over the 40-second recording, which may not be able to distinguish periods of central tremor from periods of mechanical-reflex oscillations; thus, the sensitivity and specificity of weight loading may be low in cases of very mild intermittent central tremor (e.g., mild essential tremor), especially when there is a 8–12 Hz component that can occur in enhanced physiological tremor (Deuschl et al., 2001). In such cases, time–frequency spectral analysis could be used to distinguish these tremor types. Similarly, in the analysis of functional tremor, the coherence magnitude and frequency resolution depend on the amount of spectral smoothing, and if the tremor is low-amplitude and intermittent, spurious values may occur. While proposed electrophysiological criteria exist for functional tremor (Schwingenschuh et al., 2011, Schwingenschuh et al., 2016)**,** independent validation is lacking, and the coherence results are analysis dependent. Further, since we only performed entrainment and contralateral ballistic maneuvers if functional tremor was included in the differential diagnosis by the referring provider, we could have underestimated the number of functional tremor diagnoses.

Preliminarily, electromyography and accelerometry testing appears to be useful for guiding short-term management of tremor, but we acknowledge that follow-up has not been preplanned in our study. Follow-up is warranted to determine how this procedure impacts long-term outcomes. In a broader context, the electrodiagnostic procedures applied in this study are not widely available in clinical practice due to the specific technical expertise and machines required for their implementation. While other more widely available technologies, such as smartphone accelerometers, can be used in a similar fashion (Longardner et al., 2019), several important limitations exist. The currently available smartphone applications can only assess tremor frequency and not EMG activity, and cannot be combined with other sensors (e.g., EEG) or video recordings. Furthermore, only one limb can be studied at a time, so coherence analyses cannot be performed.

In contrast with most of the published reports describing the utility of neurophysiology for movement disorders, which originate from well-established neurophysiology laboratories (Everlo et al., 2022, Grippe and Chen, 2023, Jackson et al., 2021), our institution only recently started using this technology for movement disorders within the last several years. This protocol demonstrates a framework for establishing a movement disorders clinical neurophysiology laboratory from the beginning. The work conducted in this study can readily be translated to other centers, requiring standard EMG/NCS equipment, accelerometers (e.g., Kistler), publicly available software (Elble and McNames, 2016, Vial et al., 2019), and staff with sufficient time and resources to allocate. Going forward, we hope to expand access to equipment and training for clinical electrophysiological evaluation of movement disorders to other institutions (not limited to tertiary referral centers). To address this gap, the Movement Disorders Society has developed a Clinical Neurophysiology Special Interest Group whose mission is to educate neurologists on how to implement neurophysiological procedures into clinical practice for movement disorders evaluation. While the utility of clinical neurophysiology in the evaluation of movement disorders has been described for decades (Deuschl et al., 1996), the integration of electrophysiological techniques into routine clinical care for movement disorders remains underutilized. A recent survey by the Movement Disorders Clinical Neurophysiology Task Force (Kassavetis et al., 2024) found that only 58 % of Pan-American respondents had access to clinical neurophysiology for jerk-like movements – lower than in Europe or Asia/Oceania – despite most respondents being from academic centers. This gap is also reflected in the lack of dedicated reimbursement codes for movement disorders-related neurophysiology. A movement disorders electrophysiological test protocol including surface EMG and accelerometers as we performed in this study would be reimbursed using CPT code 96002, which has a Medicare reimbursement rate of only $20.97 (Codify by AAPC, 2025). Future studies should determine how electrodiagnostic testing impacts patient outcomes longitudinally in a variety of movement disorders and should explore the cost-effectiveness of these procedures to understand how these results add value to the health care system. We hope that our work contributes to the broader effort of translating established neurophysiological methods from academic or research contexts into accessible, routine clinical care.

## Declaration of Competing Interest

The authors declare that they have no known competing financial interests or personal relationships that could have appeared to influence the work reported in this paper.
